# The estimation of protein equivalents of total nitrogen in Chinese CAPD patients: an explanatory study

**DOI:** 10.1080/0886022X.2021.2014886

**Published:** 2022-01-27

**Authors:** Chunyan Su, Tao Wang, Peiyu Wang, Xinhong Lu, Wen Tang

**Affiliations:** a Division of Nephrology, Peking University Third Hospital, Beijing, China; b Department of Nutrition and Food Hygiene, School of Public Health, Peking University, Beijing, China

**Keywords:** Urea nitrogen appearance (UNA), dietary protein intake (DPI), protein equivalent of total nitrogen appearance (PNA), continuous ambulatory peritoneal dialysis (CAPD)

## Abstract

**Objective:**

The protein equivalent of total nitrogen appearance (PNA) formula, based on the urea nitrogen appearance (UNA), is popularly used by stable continuous ambulatory peritoneal dialysis (CAPD) patients to estimate dietary daily protein intake (DPI). However, we found that the estimated DPI was higher than that directly evaluated from the dietary records of most of our CAPD patients. Therefore, in the present study, we tried to determine possible bias in PNA estimation by UNA with a nitrogen balance study of our CAPD patients.

**Methods:**

Thirty-one CAPD patients with stable clinical conditions were included. Their 3-day dietary records were reviewed by a dedicated dietitian to calculate their energy, protein, and nitrogen intake (NI). The nitrogen removal (NR) from urine and dialysate was measured by the Kjeldahl technique. Then, we calculated the proportion of urea nitrogen appearance (UNA) in total nitrogen appearance (TNA) and analyzed the possible factors that could affect this proportion.

**Results:**

Among these patients, 17 males and 14 females, the mean age was 64.19 ± 12.42, and the dialysate drainage volume was 6700 (2540) ml/day. The percentage of UNA in TNA was 63.22 ± 6.66%. Compared with the other classic nitrogen balance studies in the CAPD population, the protein nitrogen and other nonurea nitrogen losses in this study were all lower. Based on these 31 nitrogen balance studies, we proposed a pair of new equations to estimate PNA by UNA. (1) PNA = 9.3 + 7.73 UNA; (2) PNA = PNPNA + TPL = 6.7 + 7.28 UNA + TPL.

**Conclusion:**

Our study suggested that the PNA formula generated from previous European studies overestimated DPI in our CAPD patients.

## Introduction

As one of the main renal replacement therapies for patients with end-stage renal disease (ESRD), peritoneal dialysis (PD) has developed rapidly in recent years [1], especially in Asia [2]. The requirements and utilization of different nutrients change significantly in ESRD patients, and these changes ultimately place patients at higher risk for nutritional and metabolic abnormalities [3–5]. Nutritional and dietary management is therefore essential for optimal care of patients on PD. Medical staff need to evaluate dietary intake regularly in PD patients and guide them in modifying their diets accordingly to reduce malnutrition, other complications (such as volume overload, hyperphosphatemia, hypokalemia, etc), and mortality.

In terms of daily protein intake (DPI) assessment, several guidelines preferred using 3-day dietary records; the other methods, including 24-h food recalls, food frequency questionnaires, and protein equivalent of total nitrogen appearance (PNA), were considered as alternative methods [6–8]. PNA is a common tool used to estimate protein intake and is calculated using urea clearance from 24-h urine and dialysate collection in PD patients. It has been demonstrated that there is a linear relationship between the urea nitrogen appearance (UNA, the urea nitrogen output in urine and dialysate) and the total nitrogen appearance (TNA, the nitrogen output in urine, feces, and dialysate) [9,10]. TNA × 6.25 is considered to represent PNA. Hence, after determining the relationship between UNA and TNA, equations can be derived to estimate PNA from UNA. In patients who are metabolically stable, PNA reflects DPI. It is a simple and easily available method to estimate DPI. Several equations generated from a series of nitrogen balance (NB) studies on European patients undergoing continuous ambulatory peritoneal dialysis (CAPD) [9,11,12] were recommended by the international guidelines to calculate PNA [6,7], especially new Bergstrom formulas [12].

However, are these equations derived from studies on European CAPD patients suitable for Chinese patients? In our clinical practice, we found that DPI estimated by the Bergstrom formula was mostly higher (about 0.1 g/kg/day) than that directly evaluated from 3-day dietary records in our stable CAPD patients [13]. What is the reason behind this phenomenon? As we know, TNA includes UNA and NUN (non-urea nitrogen appearance). Theoretically, UNA will be influenced by protein intake, and NUN may be affected by residual renal function, dialysis dose, and membrane permeability. Thus, these equations generated from previous European studies that enrolled patients with higher DPI and dialysis doses may overestimate PNA in our CAPD patients.

Therefore, in the present study, we explored the possible bias of the generally acceptable equations for estimating PNA from UNA by analyzing nitrogen balance in our stable CAPD patients and formulated new equations.

## Methods

### Patient selection and data collection

It was a reanalysis of nitrogen balance data which had been partly published before [14]. Stable CAPD patients from our PD program were selected for this study. Eligible patients were those who (1) had been on CAPD for at least 6 months; (2) were over 18 years of age; (3) had no new complications in the previous 3 months; and (4) were willing to participate in the study. Exclusion criteria were patients who were hospitalized, bedridden, hypercatabolic (from cancer, chronic infection, hyperthyroidism, etc.) or hyperanabolic (pregnant, breastfeeding, rehabilitating after an operation, etc.), or who had psychological or cognitive disorders were not included in the present study. All patients were delivered with lactate-buffered glucose PD solutions (1.5% or 2.5% glucose) and the twin-bag connection system (Baxter Healthcare, Guangzhou, China). The study was approved by the ethics committee of Peking University Third Hospital (IRB 2021-516-02), informed consent was waived because of the retrospective design of this study. The data including patient records and information were anonymized and de-identified prior to analysis. Demographic data, including age, sex, underlying renal disease, and presence of diabetes mellitus (DM), were obtained from patients’ medical records.

### Dietary intake record and evaluation of nutritional status

In our PD program, all the patients were asked to visit our PD clinic monthly with their 3-day dietary records. The dietary data were uploaded into a computer, and dietary intake (DPI, daily protein intake; DEI, daily dietary energy intake; NI, daily nitrogen intake) were then calculated using dedicated software (PD Information Management System, Peritoneal Dialysis Center, Peking University, Beijing, China). Energy derived from dialysate glucose absorption (DGA) was also calculated as the difference between the amount of glucose in the fresh dialysis fluid and the amount in the dialysate drained. Then, DPI and TEI (total energy intake, DEI plus energy from DGA) were normalized (normalized DPI, nDPI, and normalized TEI, nTEI) by ideal body weight, which was defined as (48 kg for 150 cm) + 1.1 kg/cm for men and (45 kg for 150 cm) +0.9 kg/cm for women with Hammond’s equation [15].

Patients’ nutritional statuses were assessed by subjective global assessment (SGA). SGA is a well-validated tool to assess nutritional status based on the concept of medical history and physical examination. Patients’ nutritional status was graded as A, B, or C, reflecting normal nutrition, mild and moderate malnutrition, or severe malnutrition, respectively [16].

### Dialysis adequacy and nitrogen balance

The glucose concentration and volume of instilled dialysate 1 day before the clinic visit were recorded. Additionally, a 24-h dialysate sample was collected. Urea, creatinine, glucose, and protein in 24-h dialysate and urine were simultaneously examined. Weekly total Kt/V urea was calculated using standard methods.

The nitrogen content of 24-h dialysate (DN) and 24-h urine (UN) was determined by the Kjeldahl technique (Buchi Kjedahl Digestion and Distillation Apparatus, Switzerland). Fecal nitrogen (FN) was calculated as 0.0155 g/kg of body weight/day [17].

### Calculations

The classical NB was calculated with the equation NB = NI – TNA = NI – DN – UN − FN. Nonprotein nitrogen appearance (NPNA) was calculated by subtracting total protein loss (TPL)/6.25 from TNA. The PNA was calculated as TNA multiplied by 6.25 and PNPNA (the protein equivalent of NPNA) as 6.25 NPNA. The UNA was calculated from the sum of urea nitrogen output in urine and dialysate. Total miscellaneous nitrogen (Nmc) was calculated by subtracting protein nitrogen (Npr) and urea nitrogen appearance (UNA) from total nitrogen output in urine and dialysate: Nmc = DN + UN – UNA − Npr.

### Blood chemistries

Fasting blood samples were taken at the clinic. Serum urea, creatinine, phosphate, potassium, sodium, albumin, and carbon dioxide were measured by routine laboratory procedures.

### Statistical analysis

Statistical analysis was performed using SPSS for Windows software, version 16.0 (SPSS Inc., Chicago, IL, USA). All data were expressed as the mean ± SD or Median (interquartile range) for continuous variables according to their distributions, or percentages for categorical variables. Student’s *t*-test or Mann–Whitney *U*-test (according to the variables’ distributions) was used to compare the nitrogen clearance difference between different groups (low permeability group vs high permeability group). Linear regression was used to explore the correlation between DPI and UNA/TNA, UNA and TNA (PNA), and UNA and NPNA (PNPNA). All probabilities were two-tailed, and the level of significance was set at 0.05.

## Results

### Demographic characteristics of the study population

A total of 31 CAPD patients were included in this study: 17 males and 14 females. The primary renal diseases were diabetic nephropathy (11 cases, 35.5%), hypertensive nephropathy (8 cases, 25.8%), glomerulonephritis (7 cases, 22.6%), and others (5 cases, 16.2%). Among them, there were 5 cases with low-dose dialysis (4 l/day) whose data had been published before [14], 9 who were relatively heavy individuals with a BMI > 25, and 15 anuric cases. Demographic information about the study population is shown in Table 1.

**Table 1.** Demographic data of study population.

	Median	Mean	Sd	Range
Age (years)	68	64.19	12.42	31–81
Dialysis duration (months)^a^	32.23	32.87	24.91	6–127
BMI	22.06	23.68	3.48	18.85–30.49
Body weight (kg)	58.70	61.60	11.73	45–89
Height (cm)	162.00	160.95	7.93	146–173

^a^Non-parametric variables.

### Dialysis adequacy, dietary intake, and nutritional status in 31 CAPD patients

The median dialysate drainage volume was 6700 (2540) ml/day with Kt/V 1.65 ± 0.37 and BUN 20.86 ± 3.04 mmol/l. There were no obvious abnormalities in serum potassium, sodium, calcium, or phosphorus. The mean DPI was 45.35(11.63) g/day (nDPI 0.78 ± 0.15 g/kg/day), while the nDPI of 4 patients reached 1.0 g/kg/day. The average nTEI was 29.32 ± 5.84 kcal/kg/day, and the nTEI of 14 patients (45.2%) exceeded 30 kcal/kg/day. All patients were assessed for accepted nutritional status by SGA (2 cases with mild malnutrition). Details are shown in Table 2.

**Table 2.** Dialysis adequacy, dietary intake, and nutritional status.

	Median	Mean	SD	Minimum	Maximum
Dialysis adequacy					
Dialysate infusion volume^a^	6000.00	6525.81	1455.10	4000	8000
Dialysate drainage volume (ml/24 h)^a^	6700.00	7070.81	1568.52	4200	9100
Ultrafiltration volume (ml/24 h)	540.00	545.00	307.98	−40	1100
Urine volume (ml/24 h)^a^	80	225.48	296.78	0	1100
Total fluid removal (ml/24 h)	800	770.48	284.36	160	1280
Total sodium removal (g/day)	1.85	1.92	0.94	−0.16	3.73
Serum urea (mmol/l)	20.90	20.86	3.04	15.3	27
Serum creatinine (ummol/l)	898.00	944.45	256.82	515	1460
CO_2_ CP (mmol/l)	27.50	27.69	3.24	21.4	33.7
Serum phosphorus (mmol/l)	1.71	1.65	0.34	0.89	2.23
Serum potassium (mmol/l)	4.19	4.16	0.56	3.0	5.0
Serum sodium (mmol/l)	139.00	138.35	4.31	128	146
Kt/V	1.68	1.65	0.37	0.90	2.66
rKt/V^a^	0.06	0.22	0.32	0	1
Dietary intake and nutrition status					
DPI (g/day)^a^	45.35	45.36	11.07	25.42	79.59
nDPI (g/kg/day)	0.76	0.78	0.15	0.53	1.13
DEI (kcal/day)	1407.04	1388.04	291.44	808.88	1991.96
Energy from DGA (kcal/day)^a^	278.17	296.82	80.01	166.04	510.64
nTEI (plus dialysate, kcal/kg/day)	27.96	29.32	5.19	20.06	44.52
PNA (g/day)	43.29	44.31	10.85	23.63	74.14
nPNA (g/kg/day)	0.73	0.76	0.14	0.50	1.17
Fat (g/day)	51.30	52.19	16.68	12.60	88.16
Carbohydrate (g/day)	188.08	194.94	54.29	109.6	339.6
Potassium intake (mg/day)	1301.10	1304.40	387.86	687.90	2414.63
Phosphorous intake (mg/day)	654.79	680.63	177.05	367.25	1191.35
Serum albumin (g/l)	41.00	40.03	3.58	32	47
Hemoglobin (g/l)	113.5	113.63	10.95	95	142

CO2 CP, carbon dioxide combining power; rKt/ V, renal Kt/ V; DPI, daily protein intake; nDPI, normalized DPI; DEI, daily energy intake; DGA, dialysate glucose absorption; nTEI, normalized TEI; PNA, protein nitrogen appearance; nPNA, normalized PNA.

^a^Non-parametric variables.

### Nitrogen balance in 31 patients

The 31 CAPD patients maintained a neutral nitrogen balance (NB 0.17 ± 1.11 g/day, with NI 7.26 (1.86) g and TNA 7.09 ± 1.74 g/day). The median of total protein loss was 4.39(1.50) g/day, which was equivalent to 0.70 (0.24) g/day of nitrogen. The average NPNA was 6.34 ± 1.62 g/d (89.08 ± 3.36% of TNA). The average UNA was 4.52 ± 1.33 g/day (63.22 ± 6.66% of TNA). The loss of Nmc accounted for 12.03% of TNA. Detailed information on the nitrogen balance is shown in Supplementary Table 1.

### Factors affecting UNA percentage

The D-UNA/DN (UNA proportion in dialysate) of 31 dialysate solution samples was lower than the U-UNA/UN (UNA proportion in urine) of 16 urine samples (0.72 ± 0.08 vs 0.79 ± 0.12, *t* = 2.104, *p* = 0.047).

To analyze the influence of membrane permeability on UNA/TNA, we calculated the D/PCr24 h (24-h dialysate creatinine/plasma creatinine) of each patient and then used the means of D/PCr24 h in different dialysate bags groups (4 l, 6 l, and 8 l) as cutoff value to divide the patients into two groups, named the low D/P group (low permeability, 17 cases) and the high D/P group (high permeability, the other 14 cases). There were no significant differences in UNA of dialysate (D-UNA) or total nitrogen of dialysate (DN) between the two groups. Protein loss in the high D/P group was higher than that in the low D/P group, 4.52(0.98) g/day vs 3.43(2.15) g/day, but the difference did not reach statistical significance (*p* = 0.128) which was probably due to the small sample size. D-UNA/DN in the high D/P group was significantly lower than that in the low D/P group (0.70 ± 0.08 vs 0.75 ± 0.07, *p* = 0.050). See Table 3 for details.

**Table 3.** The comparison of nitrogen clearance between patients with different membrane permeability.

	Low D/P (*n* = 17)	High D/P(*n* = 14)	*t*/*U*	*p*
DPL (g/day)^a^	3.43 (2.15)	4.52 (0.98)	1.548	0.128
D-UNA (g/day)	4.02 ± 0.84	3.66 ± 1.00	1.072	0.293
DN (g/day)	5.39 ± 1.17	5.27 ± 1.31	0.272	0.787
D-UNA/DN	0.75 ± 0.07	0.70 ± 0.08	2.047	0.050

DPL, dialysate protein loss; D-UNA, urea nitrogen appearance in dialysate; DN, nitrogen in dialysate.

^a^Using Mann–Whitney *U*-test, expressed as Median (Interquartile Range).

**Table 4.** The abbreviations list.

Abbreviations	Full names	Explanations
DPI	Daily protein intake	
nDPI	Normalized DPI	Normalized DPI by ideal body weight
DEI	Daily dietary energy intake	
DGA	Dialysate glucose absorption	Glucose absorbed from dialysate
TEI	Total energy intake	DEI plus energy from DGA
nTEI	Normalized TEI	Normalized TEI by ideal body weight
NI	Daily nitrogen intake	NI = DPI/6.25
UNA	Urea nitrogen appearance	The urea nitrogen output in urine and dialysate
TNA	Total nitrogen appearance	Total nitrogen output in urine, feces, and dialysate
PNA	Protein equivalent of total nitrogen appearance	Protein equivalent of TNA
nPNA	Normalized PNA	Normalized PNA by ideal body weight
NB	Nitrogen balance	NB = NI-TNA
NUN	Non-urea nitrogen appearance	NUN = TNA-UNA
DN	Dialysate nitrogen	Nitrogen content of 24-h dialysate
UN	Urine nitrogen	Nitrogen content of 24-h urine
FN	Fecal nitrogen	
TPL	Total protein loss	Protein loss from dialysate and urine
DPL	Dialysate protein loss	Protein loss from dialysate
NPNA	Nonprotein nitrogen appearance	NPNA = TNA − TPL/6.25
PNPNA	The protein equivalent of NPNA	PNPNA = 6.25 NPNA
Nmc	Miscellaneous nitrogen	Nmc = DN + UN – UNA − Npr
Npr	Protein nitrogen	Npr = TPL/6.25
UNA/TNA		UNA percentage in TNA
D-UNA/DN		UNA proportion in dialysate nitrogen
U-UNA/UN		UNA proportion in urine nitrogen

The DPI (g/day) was positively correlated with UNA/TNA (*r* = 0.356, *p* = 0.049, by Spearman correlation analysis, see Supplementary Figure 1). However, the nDPI (g/kg/day) was not correlated with UNA/TNA (*r* = 0.217, *p* = 0.240). Univariate Spearman correlation analysis showed that UNA/TNA was negatively correlated with DPL (dialysate protein loss, *r* = −0.416, *p* = 0.020) and TPL (*r* = −0.383, *p* = 0.033).

### The relationship between UNA and TNA(PNA), UNA and NPNA(PNPNA)

**Figure 1.**
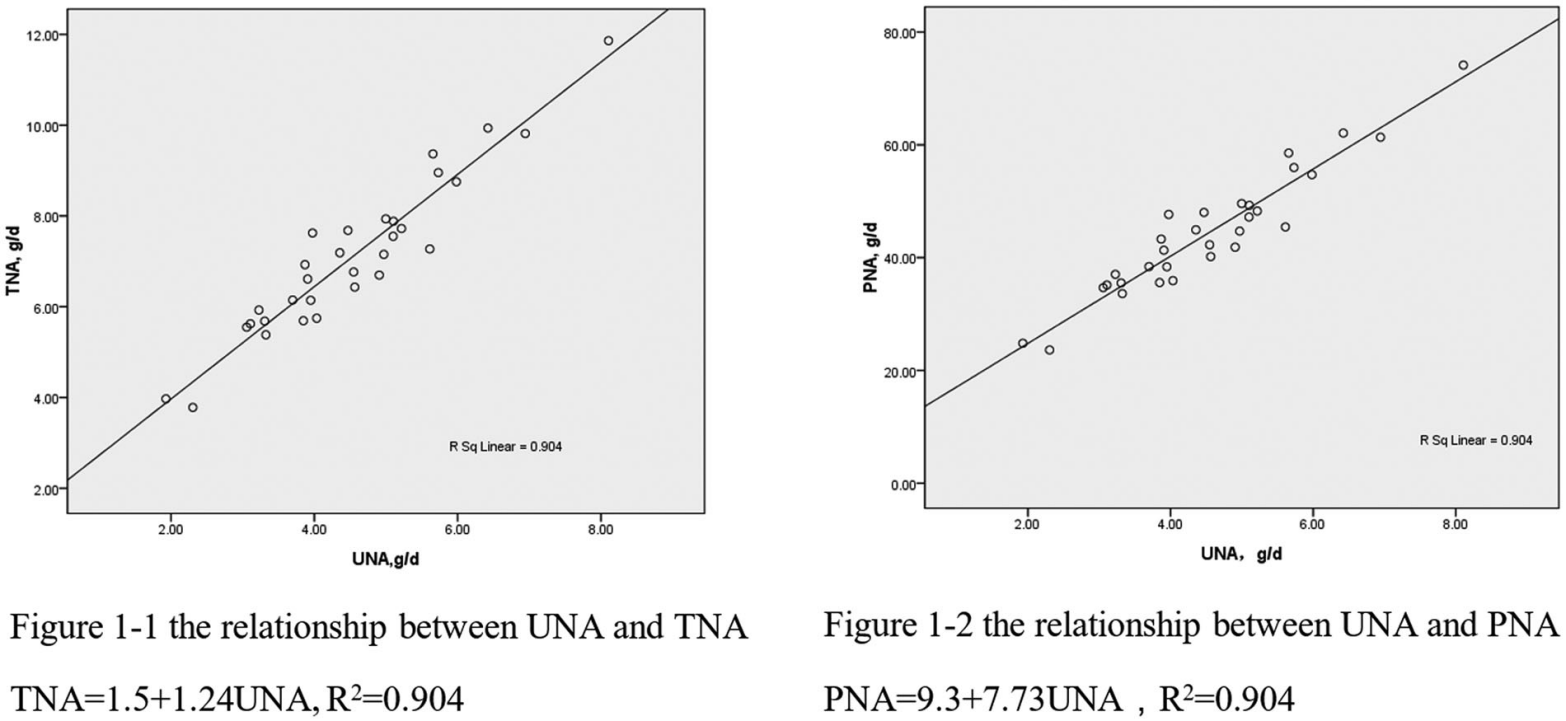
The relationship between UNA and TNA (PNA). UNA: urea nitrogen appearance; TNA: total nitrogen appearance; PNA: protein equivalent of nitrogen appearance.

**Figure 2.**
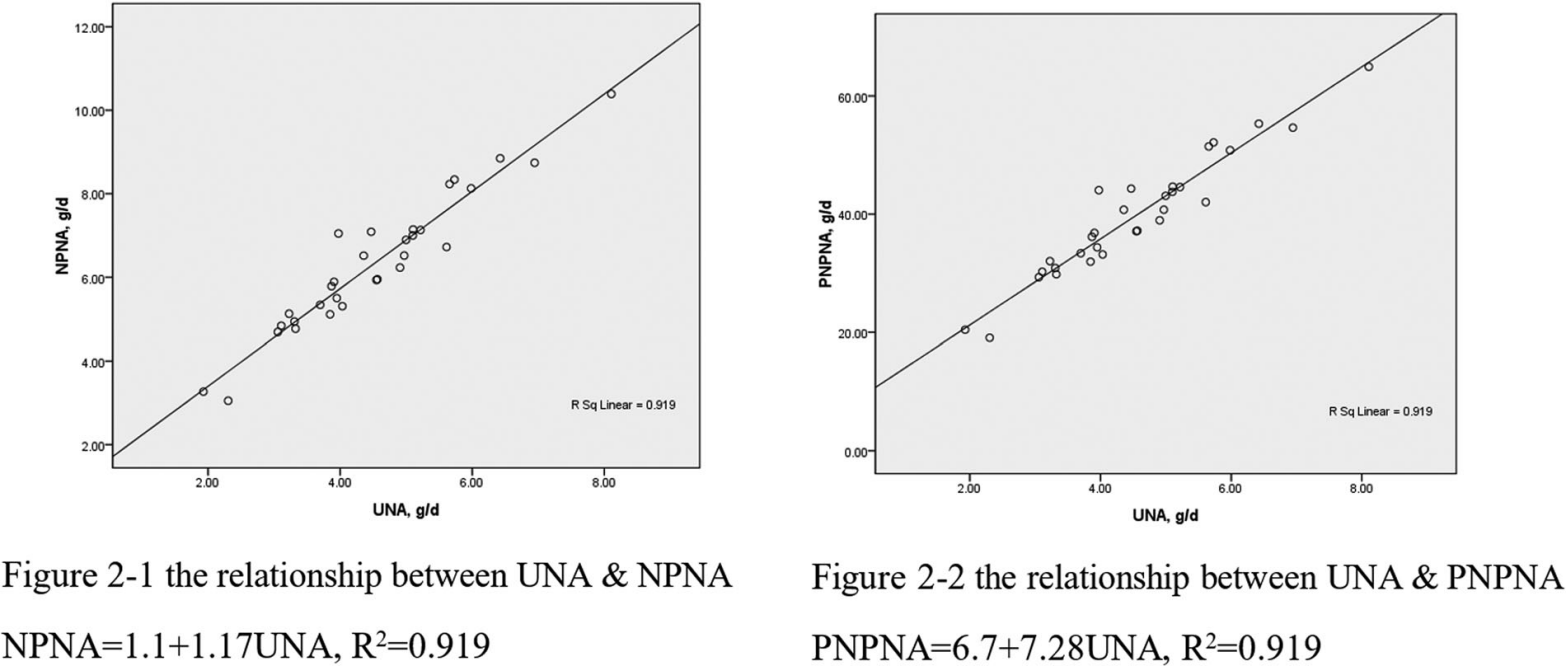
The relationship between UNA and NPNA (PNPNA). UNA: urea nitrogen appearance; NPNA: non-protein nitrogen appearance; PNPNA: protein equivalent of non-protein nitrogen appearance

The relationships of UNA and TNA (PNA) are shown in Figure 1-1 and 1-2. UNA correlated positively with TNA (PNA), *r* = 0.951, *p* < 0.001. The linear regression equations between UNA and TNA (PNA) are:

$$\text{TNA}=1.5+1.24\;\text{UNA}\;(R^{2}=0.904,\;p<0.001).\text{PNA}=9.3+7.73\;\text{UNA}\;(\text{new}\;\text{formula}\;1),\;(R^{2}=0.904,\;p<0.001).$$

The relationships of UNA and NPNA (PNPNA) are shown in Figure 2-1 and 2-2. UNA was also positively correlated with NPNA (PNPNA), *r* = 0.959, *p* < 0.001. The linear regression equations between UNA and NPNA (PNPNA) are:

$$\text{NPNA}=1.1+1.17\;\text{UNA}\;(R^{2}=0.919,p<0.001)\text{PNPNA}=6.7+7.28\;\text{UNA}\;(R^{2}=0.919,p<0.001)\text{PNA}=\;\text{PNPNA}\;+\text{TPL}=6.7+7.28\;\text{UNA}+\text{TPL}\;(\text{new}\;\text{formula}\;2)$$

## Discussion

In the present study, based on 31 nitrogen balance studies of stable Chinese CAPD patients, we derived a pair of new formulas for calculating PNA:

$$\begin{array}{l} \text{PNA}=\text{9}.\text{3}+\text{7}.\text{73 UNA }\left(\text{new formula 1}\right),\\ \text{PNA}=\text{ PNPNA}+\text{TPL}=\text{6}.\text{7}+\text{7}.\text{28 UNA}+\text{TPL }\\ \left(\text{new formula 2}\right). \end{array}$$

From the basic structure of the formula, the new formula is quite different from other formulas recommended by international guidelines [6]. The guidelines recommend several PNA formulas. The first is the Bergstrom formula [12].

Formula 1: PNA (g/day) = 20.1 + 7.50 UNA (g/day)

Formula 2: PNA (g/day) = 15.1 + 6.95 UNA       (g/day) +TPL

Another is the K-B formula [10]

Formula 1: PNA (g/day) = 34.6 + 5.86 UNA (g/day)

Formula 2: PNA (g/day) = 22.5 + 6.16 UNA (g/day)       + TPL

The differences between the new formula and other formulas consist of the intercept and UNA coefficient. This indicates that the proportion of NUNs in the present study population was small, and the variation in TNA was more influenced by UNA. In this study, the average UNA was 4.52 ± 1.33 g, accounting for 63.22 ± 6.66% (49 ∼ 77%) of TNA and 70.91 ± 6.23% of NPNA. This result was higher than that in Bergstrom’s NB study (UNA/TNA, 58.1%) [9], higher than that of CAPD patients with DPI 1 g/kg/day in Blumenkrantz’s NB studies (54.6%), lower than that of patients with DPI 1.4 g/kg/day (68.1%) [10], and lower than that of pre-dialysis population (68.1%) [18], see Supplementary Figure 2. It was the result of a combination of many influencing factors.

First, the influence of DPI. Phansalkar et al. found that normal adults who had a high DPI had an increased proportion of UNA. When the DPI was 100 g/day, 75 g/day, and 44 g/day, the proportions of UNA were 88%, 82%, and 67%, respectively [19]. Blumenkrantz et al. reported that the UNA percentage was 68.1% when the DPI was 1.4 g/kg/day but only 54.6% when the DPI was 1.0 g/kg/day in NB studies of their CAPD population [10]. The present study also found that DPI (g/day) was positively correlated with UNA/TNA (*r* = 0.356, *p* = 0.049). This is mainly because urea nitrogen is the final product of dietary protein metabolism. When dietary protein content is high, more urea nitrogen is produced. On the other hand, NUNs have little relationship with DPI, so the proportion of UNA will increase. In this study, the average nDPI was only 0.78 g/kg/day (45.36 g/day), and UNA/TNA should be lower than that in Bergstrom and Blumenkrantz’s studies. However, the average UNA/TNA ratio in this study (63.22 ± 6.66%) was higher than that of patients with DPI at 1.0 g/kg/day and 1.3 g/kg/day in the aforementioned studies and only lower than that of patients with DPI at 1.4 g/kg/day [9,10]. Therefore, although DPI is positively correlated with UNA/TNA, this factor is not the reason why UNA/TNA is higher in this study than in other NB studies.

Second, the influence of protein loss. Total protein loss (TPL) includes protein loss from residual kidney and peritoneal dialysis, but dialysate protein loss is most significant. In the present study, the proportion of UNA in urine (0.79 ± 0.12) was higher than that in dialysate fluid (0.72 ± 0.08), *p* = 0.047. The primary reason lies in the difference in protein loss between the residual kidney and peritoneal dialysis. The 24-h TPL was 4.39 (1.50) g/day, which was significantly lower than in the results of other studies. In the classic NB studies of CAPD patients, Bergstrom et al. reported an average TPL of 7.0 ± 2.1 g/day [9], while Blumenkrantz et al. reported a TPL of 9.2 ± 0.6 g/day [10]. Dulaney et al. summarized protein loss in CAPD patients and obtained similar results [20]. The low TPL may be one of the main reasons why the intercept in new formula 1 is smaller than that in other formulas.

A high protein clearance rate reflects high peritoneal permeability. Many studies have shown that protein clearance is associated with volume overload and chronic inflammation in stable PD populations [21–23]. High glucose solution and its degradation products can cause chronic inflammatory reactions and peritoneal fibrosis. Hassan et al. [23] found that glucose load in PD patients was positively correlated with volume overload and hypersensitive CRP and IL-6 levels. Yung et al. [24] reported that low glucose solution could protect peritoneal integrity and reduce fibrosis and inflammation. Although only glucose-based PD solution is currently available in China, the first step for volume control in our clinical practice is to educate patients about restricting water and salt intake, so as to minimize the use of high glucose solution to protect peritoneal function. This clinical practice may also reduce chronic inflammation in the peritoneum and reduce protein loss in the dialysate. Interestingly, in recent years, several studies also reported 4–6 g/day dialysate protein loss, which was close to our data [25,26]. This may indicate that the clinical PD practice in recent years has changed considerably, and reducing high glucose solution use, volume control, and inflammatory status control can reduce protein loss.

Finally, the influence of other miscellaneous nitrogen. In this study, the average loss of Nmc, such as nitrogen from creatinine, uric acid, amino acids, and peptides, was approximately 0.014 g/kg/day, accounting for 12.83% of TNA. This coefficient is similar to that of predialysis patients (mean 0.0155 g/kg/day) [18], much lower than the other two NB studies of the CAPD population [9,10]. Figure 1 shows the composition of TNA in different NB studies, which indicate that the TNA and UNA/TNA in this study are relatively similar to those in Maroni’s study [18]. The Nmc is lower than that reported in previous NB studies of the CAPD population, which is another major reason for the relatively high UNA/TNA ratio in this study and the main reason for the intercept in new formula 2, which is smaller than that in other formulas.

Why is there less Nmc in this study population? Our clinical practice, which differs from that in the West, may play an important role. In our clinical practice, we adopted incremental dialysis which was proved to have the equal or even better outcome as compared to conventional dialysis [27], and educated patients on controlling water and salt intake to minimize high glucose solution used for volume balance. The dialysate drainage volume and ultrafiltration were generally small (6700 [2540] ml/day and 545.00 ± 307.98 ml/day, respectively) in the present study. However, the dialysate infusion volume (approximately 8–9 l/day) and ultrafiltration (approximately 2 l/day) in classical NB studies [9,10] were significantly higher than those in the present study. The principles of PD for solute removal mainly include diffusion and convection. Small molecule solutes, such as creatinine, uric acid, and amino acids, are mainly cleared by diffusion, which is closely related to the dialysis dose. Medium molecular toxins such as peptides are mainly removed by convection. In Bergstrom and Blumenkrantz’s study [9,10], the dialysate infusion volume and ultrafiltration were both higher, and more solutes were cleared by diffusion and convection. Therefore, it is not difficult to understand why the Nmc in their studies was relatively high. The most recent study, which mainly enrolled APD patients with even higher dialysis doses compared with previous Bergstrom and Blumenkrantz’s studies, reported a UNA/TNA ratio of only 45% [28]. It also proved the influence of dialysis dose on NUN clearance.

In addition, the 31 CAPD patients had typical Asian body sizes (BMI 23.68 ± 3.48), body weights (61.60 ± 11.73 kg), and heights (160.95 ± 7.93 cm), all lower than those of patients in the West. Their dialysate drainage volume (4.2–9.1 l/day), NI (4.07 ∼ 12.73 g), and TNA (3.78 ∼ 11.86 g) were widely representative. The dialysate drainage volume and ultrafiltration were relatively small, which may be the main reason for the difference between the new formula and others, and it is also in line with the characteristics of CAPD clinical practice in Asia [29–31] (generally 3–4 bags/day of 2 l solution, with approximately 1 l/day ultrafiltration). For these reasons, the new formulas may be more suitable for the estimation of DPI in Asian CAPD populations than other formulas based on NB studies in Western populations.

There were several limitations of the present study. First, we did not directly determine the nitrogen of dietary intake. Previous studies have implicated that patients’ 3-day dietary records checked by a dedicated dietitian using food models are acceptable for estimating DPI [32,33]. Second, we did not directly determine the fecal nitrogen loss. Studies by Masud T. et al. have reported that NUN in predialysis patients varies with body weight (0.031 g/kg/day) [16], which is widely used to estimate NUN in predialysis patients. However, in the present study, we measured the nonurea nitrogen from urine and dialysate, so we used 0.0155 g/kg/day to estimate FN from the same study [16]. Third, nitrogen loss from the integument, in exfoliated skin, sweat, and growing hair and nails, was reported by Calloway as only 149 ± 51 mg/day in normal healthy young men ingesting 75 g protein/d [34]. We ignored this part of nitrogen loss in this study. The last, the sample size was relatively small, the new formula may need further validation and verification. Other well-designed large sample studies were warranted.

## Conclusions

The present study suggested that the widely accepted PNA formula, generated with data from European PD patients, overestimated DPI in Chinese CAPD patients. In the present study, a pair of new formulas for calculating PNA were generated based on 31 nitrogen balance studies of stable Chinese CAPD patients: PNA = 9.3 + 7.73 UNA (new formula 1), PNA = PNPNA + TPL = 6.7 + 7.28 UNA + TPL (new formula 2). The new formula has a smaller intercept and a larger UNA coefficient than previous formulas recommended by international guidelines, which was mainly influenced by the lower protein loss and other miscellaneous nitrogen. Since the study population was Asian with fairly typical Asian body type and the dialysis regimen conformed to the characteristics of Asian CAPD clinical practice, the new formulas might be more suitable for DPI estimation in Asian CAPD populations.

## Supplementary Material

Supplementary Material
