# Supplementary material for: The estimation of protein equivalents of total nitrogen in Chinese CAPD patients: an explanatory study
Source: Ren Fail. 2022 Jan 27;44(1):14–22. doi: 10.1080/0886022X.2021.2014886 (PMC8815777; doi:10.1080/0886022X.2021.2014886)
Supplement: Supplementary Material [file IRNF_A_2014886_SM2964.pdf]

Supplementary material for Su et al., "The estimation of protein equivalents of total nitrogen in Chinese CAPD patients: an explanatory study", *Renal Failure*, 2021.

Supplementary Table 1: nitrogen balance studies of 31 CAPD patients

No.	NI*, g/d	DN, g/d	UN*, g/d	FN*, g/d	UPL*, g/d	DPL*, g/d	TPL*, g/d	NPNA , g/d	UNA, g/d	TNA, g/d	NB g/d
B1	7.62	6.62	0	1.10	0	3.69	3.69	7.13	5.22	7.72	-0.11
B2	6.85	5.11	1.43	1.14	0.41	3.29	3.69	7.09	4.47	7.68	-0.83
B3	7.42	6.01	0	1.18	0	4.20	4.20	6.52	4.36	7.19	0.23
B4	9.07	6.36	0	1.19	0	3.43	3.43	7.00	5.10	7.55	1.51
B5	12.73	6.09	4.53	1.25	0.39	8.81	9.20	10.39	8.11	11.86	0.87
B6	11.33	5.51	2.18	1.27	0.55	3.29	3.84	8.34	5.73	8.95	2.37
B7	6.84	4.62	1.26	1.27	0.66	3.30	3.96	6.52	4.97	7.15	-0.31
B8	9.07	4.15	3.23	1.37	0.55	3.35	3.90	8.13	5.98	8.75	0.32
L1	6.26	4.48	0.91	0.74	0.21	3.87	4.08	5.50	3.95	6.13	0.13
L2	4.07	2.81	0.41	0.76	0.10	4.33	4.43	3.27	1.93	3.98	0.09
L3	5.72	4.0	0.53	0.84	0.04	2.76	2.80	4.77	3.32	5.37	0.35
L4	5.75	4.75	0	0.79	0	5.36	5.36	4.69	3.06	5.55	0.20
L5	4.50	4.80	0	0.82	0	4.89	4.89	4.84	3.11	5.62	-1.13
A1	7.04	5.05	0	0.70	0	2.75	2.75	5.31	4.03	5.75	1.30
A2	5.27	3.06	0	0.72	0	4.56	4.56	3.05	2.30	3.78	1.49
A3	7.19	4.91	0	0.78	0	4.64	4.64	4.94	3.31	5.68	1.50
A4	7.66	7.11	0	0.83	0	6.49	6.49	6.90	5.00	7.93	-0.27
A5	7.67	6.74	0	0.88	0	3.61	3.61	7.05	3.97	7.63	0.04
A6	7.26	6.38	0	0.89	0	3.42	3.42	6.72	5.61	7.27	-0.02
A7	5.83	5.69	0	0.92	0	4.49	4.49	5.89	3.91	6.61	-0.78

A8	7.98	6.95	0	0.93	0	4.63	4.63	7.14	5.10	7.88	0.09
A9	7.71	8.41	0	0.96	0	7.10	7.10	8.23	5.66	9.37	-1.66
A10	5.92	5.97	0	0.96	0	7.13	7.13	5.79	3.87	6.93	-1.01
O1	7.41	5.36	0.55	0.85	0.24	4.94	5.19	5.93	4.55	6.76	0.65
O2	7.49	4.88	0.87	0.95	0.20	2.71	2.91	6.23	4.91	6.70	0.80
O3	6.53	4.05	1.47	0.91	0.48	2.52	3.00	5.95	4.56	6.43	0.10
O4	8.45	5.41	3.37	1.04	0.77	5.93	6.71	8.74	6.94	9.82	-1.37
O5	6.14	4.45	0.43	0.81	0.19	3.43	3.62	5.11	3.85	5.69	0.45
O6	7.78	6.32	2.74	0.88	0.42	6.38	6.80	8.85	6.42	9.94	-2.15
O7	8.74	4.91	0.13	0.89	0.05	4.95	5.00	5.13	3.23	5.93	2.82
O8	5.71	4.36	0.88	0.91	0.76	4.29	5.05	5.34	3.70	6.15	-0.44
Median	7.26	5.11	0.13	0.91	0.38	4.28	4.39	6.23	4.47	6.93	0.10
Mean	7.26	5.33	0.80	0.95	0.19	4.50	4.69	6.34	4.52	7.09	0.17
Sd	1.77	1.21	1.21	0.18	0.26	1.47	1.48	1.62	1.33	1.74	1.11
Minimum	4.07	2.80	0	0.70	0	2.52	2.75	3.05	1.93	3.78	-2.15
Maximum	12.73	8.41	4.53	1.37	0.77	8.81	9.20	10.39	8.11	11.86	2.82

NI, nitrogen intake; DN, nitrogen in 24-h dialysate; UN, nitrogen in 24-h urine; FN, fecal nitrogen; UPL, urine protein loss; DPL, dialysate protein loss; TPL, total protein loss; NPNA, Non-protein nitrogen appearance; UNA, urea nitrogen appearance; TNA, total nitrogen appearance; NB, nitrogen balance.

* Non-parametric variables

Supplementary Figure 1: the relationship between DPI and UNA/TNA

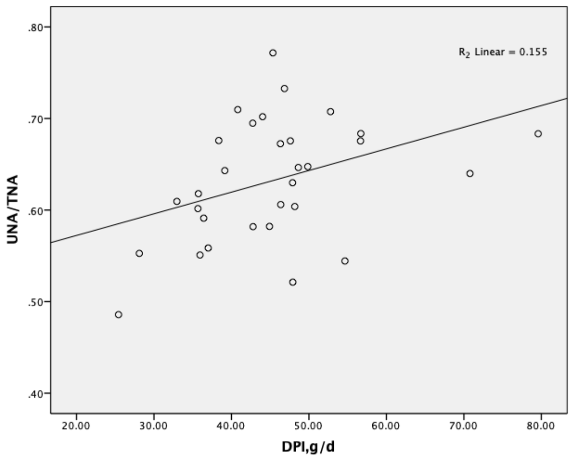

Figure 1 the relationship between DPI and UNA/TNA

UNA/TNA=0.525+0.002DPI, R^2=0.155, P=0.029

DPI, dietary protein intake; UNA/TNA, urea nitrogen appearance/total nitrogen appearance.

Supplementary Figure 2: The component of TNA among different NB studies

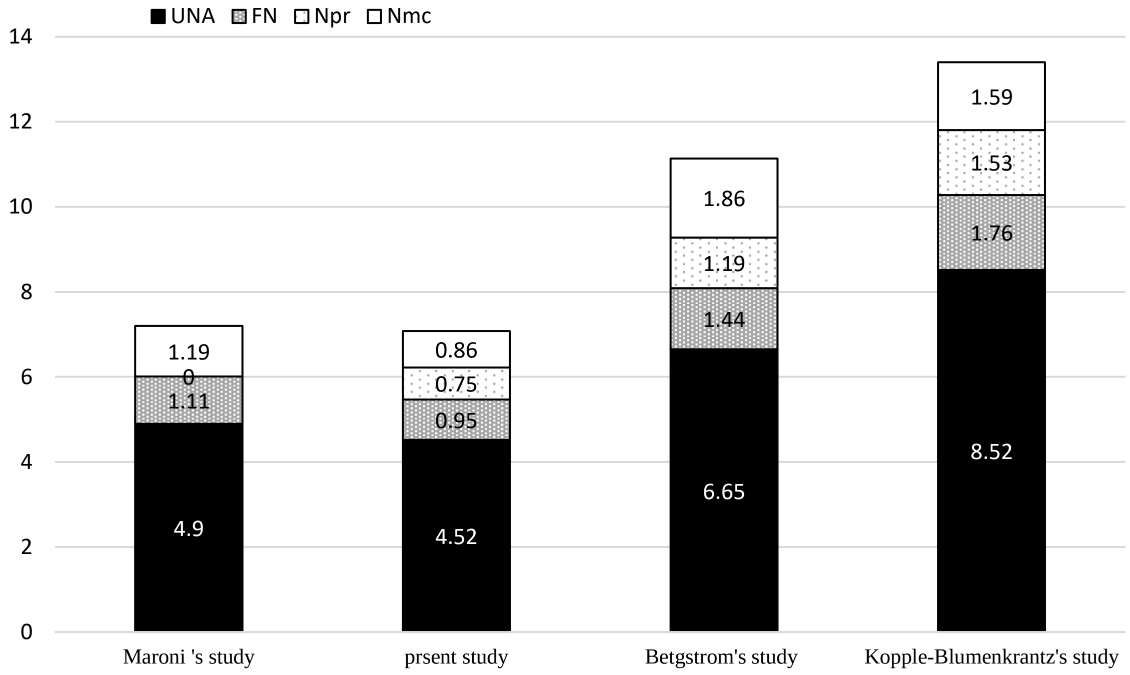

Figure 3 the component of TNA among different NB studies

UNA, urea nitrogen appearance; FN, fecal nitrogen; Npr, protein nitrogen; Nmc, miscellaneous nitrogen.

Maroni's study, pre-dialysis population, 33 NB studies, DPI 48g/d (0.64g/kg/d)

Present study, CAPD population, 31 NB studies, DPI 45g/d (0.78g/kg/d)

Bergstrom's study, CAPD population, 23 NB studies, DPI 86g/d (1.3g/kg/d)

Kopple-Blumenkrantz's study, CAPD population, 13 NB studies, DPI 94g/d (1.20g/kg/d）
